# Current state of research on acupuncture for the treatment of amyotrophic lateral sclerosis: A scoping review

**DOI:** 10.3389/fneur.2022.1019156

**Published:** 2022-11-03

**Authors:** Siyang Peng, Yukun Tian, Weiqian Chang, Yajing Yang, Shaohong Li, Jinxia Ni, Wenzeng Zhu

**Affiliations:** ^1^Acupuncture Department, Guang'anmen Hospital, China Academy of Chinese Medical Sciences, Beijing, China; ^2^Acupuncture Department, Dongzhimen Hospital of Beijing University of Chinese Medicine, Beijing, China

**Keywords:** amyotrophic lateral sclerosis, motor neuron disease, acupuncture, complementary and alternative medicine, scoping review

## Abstract

**Objective:**

To provide an overview of the range and characteristics of existing evidence, research gaps, and future research priorities in treating amyotrophic lateral sclerosis (ALS) with acupuncture.

**Method:**

Clinical studies on acupuncture treatment for ALS were searched in 9 databases and two websites. Two independent researchers screened the literature according to the inclusion and exclusion criteria; extracted the demographic data, interventions, and significant findings of the studies; and comprehensively analyzed the characteristics and limitations of the included articles.

**Results:**

A total of 2,326 studies were retrieved, of which 92 were included. Most of the studies were conducted in China, with the number increasing over time. Study designs included case reports, case series, randomized controlled trials (RCTs), and before-and-after studies, among which case reports were the most frequently used. A total of 1,388 patients were enrolled, of whom 1,031 had ALS, 274 had progressive bulbar palsy (PBP), 60 had progressive muscle atrophy (PMA), and 23 had primary lateral sclerosis (PLS). Acupuncture interventions included body acupuncture, electroacupuncture, acupoint injection, scalp acupuncture, acupoint massage, Sa-am acupuncture, needle-embedding therapy, auricular acupuncture, venom pharmacopuncture therapy, plum blossom needling, acupoint paste, electroacupuncture, and needle warming through moxibustion. The most frequently used acupoints were ST36, LI4, SP6, and LI11. Acupuncture is often applied in combination with other treatments, such as herbal or Western medicine. The frequency of treatment ranged from once a month to three times a day, and the duration of treatment ranged from 5 days to 3 years. Clinical symptoms, muscle strength, and effective rates were the most frequently used outcomes. Most studies reported significant efficacy, and only a few studies reported adverse events explicitly.

**Conclusion:**

Evidence gaps include poor study design, complex interventions, limited significance of the selected outcomes, and limited study reporting. The promotion of acupuncture treatment for ALS still faces several obstacles. Rigorous study design and conduct, standardized intervention and outcome measurements, and normative reporting are needed to investigate the efficacy and safety of acupuncture treatment for ALS.

## Introduction

Amyotrophic lateral sclerosis (ALS), also known as motor neuron disease (MND), is characterized by degeneration of both upper and lower motor neurons, leading to muscle weakness and eventual paralysis (1). Progressive neurological deterioration involves the corticospinal tract, brainstem, and anterior horn cells of the spinal cord (2). Other types of ALS include progressive bulbar palsy (PBP), primary lateral sclerosis (PLS), and progressive muscular atrophy (PMA). Death generally occurs within 2–4 years of onset owing to respiratory failure (3). The worldwide all-age prevalence is 4.5 (4.1–5.0) per 100,000 people, and the all-age incidence is 0.78 per 100,000 person-years (4). The pathophysiology of ALS remains unknown, limiting the development of disease-modifying therapies (5). The only two drugs approved for the treatment of ALS are riluzole and edaravone (6, 7). Riluzole can only prolong the median survival time of patients with ALS by ~3 months (7). It is still unclear whether edaravone therapy prolongs survival in the long term (8).

Due to the lack of effective treatments, many patients with ALS turn to complementary and alternative treatments, such as acupuncture and herbal medicine (9). The possible mechanisms of acupuncture in treating ALS mainly include: (i) glutamate excitotoxicity is one of the most important hypotheses in the pathogenesis of ALS, acupuncture can antagonize the excitotoxicity of glutamate, protect the motor neurons, and delay the progression of the disease in animal models (10, 11); (ii) acupuncture can protect the motor neurons by up-regulating the expression of autophagy-associated proteins, strengthening autophagy, and promoting the elimination of abnormal proteins in the transgenic mouse model of ALS (11); and (iii) acupuncture can suppress neuroinflammation responses to protect motor neurons and affect the apoptosis of motor neurons, for example, animal studies found that acupuncture could reduce the activity of microglial cells and the expression of TNF-α to reduce neuronal cell loss and improve motor function in ALS models (12, 13). Some clinical studies have reported that acupuncture may be an effective treatment for ALS, by relieving symptoms and improving quality of life (14). However, the evidence supporting acupuncture for ALS is unsystematic, and the credibility of these findings is limited by non-RCT study designs, unverified outcome measures, small sample sizes, or short follow-up periods. Thus, these reports cannot provide high-quality evidence for the clinical application of acupuncture in ALS treatment.

To provide an overview of the range and characteristics of any existing evidence, research gaps, and future research priorities in treating ALS with acupuncture, we conducted a scoping review to summarize and critically analyze the findings of all published articles (15). This scoping review follows the PRISMA Extension for Scoping Reviews (PRISMA-ScR) (16). The protocol was registered on the Open Science Framework (OSF) with Registration DOI https://doi.org/10.17605/OSF.IO/RC5FT.

## Materials and methods

### Identifying the review questions

Before starting this study, the broad exploratory research question was, “What has been studied about acupuncture treatments administered to patients with ALS? (17).” The more detailed research questions were as follows: What kind of research has been conducted? What are the characteristics of acupuncture intervention in clinical research? What is its efficacy and safety? What are the highlights and directions for future research?

### Literature search

The retrieval strategy followed the principle of PICO. Population: patients with ALS/MND/PBP/PLS/PMA; animal and cell studies were excluded. Intervention: the term acupuncture refers to puncturing with a needle; however, acupuncture may also involve the application of other types of stimulation at certain points (18). In our study, we included any type of commonly used acupuncture that stimulates certain points with needles, electricity, acupoint injection, or pressure. Studies that used acupuncture alone or in combination with other therapies were also included. Study design: all types of clinical studies were included, including case reports, case series, case-cohort studies, before-after studies, retrospective and prospective cohort studies, randomized controlled trials (RCTs), systematic reviews, and narrative reviews. No language or date limitations were applied (inception to October 2nd, 2022). Conference articles and abstracts, dissertations, and repeatedly published articles were excluded. The searched databases included PubMed, Cochrane Library, Web of Science (WOS), Allied and Complementary Medicine Database (AMED), Cumulative Index to Nursing and Allied Health Literature (CINAHL), China National Knowledge Infrastructure (CNKI), VIP Chinese Journal Service Platform (VIP), WanFang Data Knowledge Service Platform (WanFang), and China Biology Medicine disc (CBM). Supplementary File 1 presents the details of the electronic search strategy. Other search resources included two websites, www.alsuntangled.com and www.itmonline.org.

### Literature selection

All the retrieved articles were imported into EndNote X9.3.1. A duplication checking function was used to remove duplicate studies. Two researchers screened the titles and abstracts of the articles independently, and those that met the inclusion criteria were retained for further screening. Subsequently, two researchers read the full text of the articles and retained those that met the criteria. When they disagreed on whether to include a certain article, the two researchers discussed it before the final decision. If no agreement could be reached, a third researcher would help to evaluate whether the article should be included.

### Data extraction

The extracted data were as follows: author, year of publication, country of origin, purpose, population and sample size, methodology, intervention and comparator, acupoints, frequency and duration of the intervention, outcomes, adverse events, significant findings, and conclusion. The Excel table used for extracting the data was designed in advance. Two researchers worked independently and checked the data in real time to ensure the accuracy of the information.

## Results

### Results of the search

**Figure 1:**
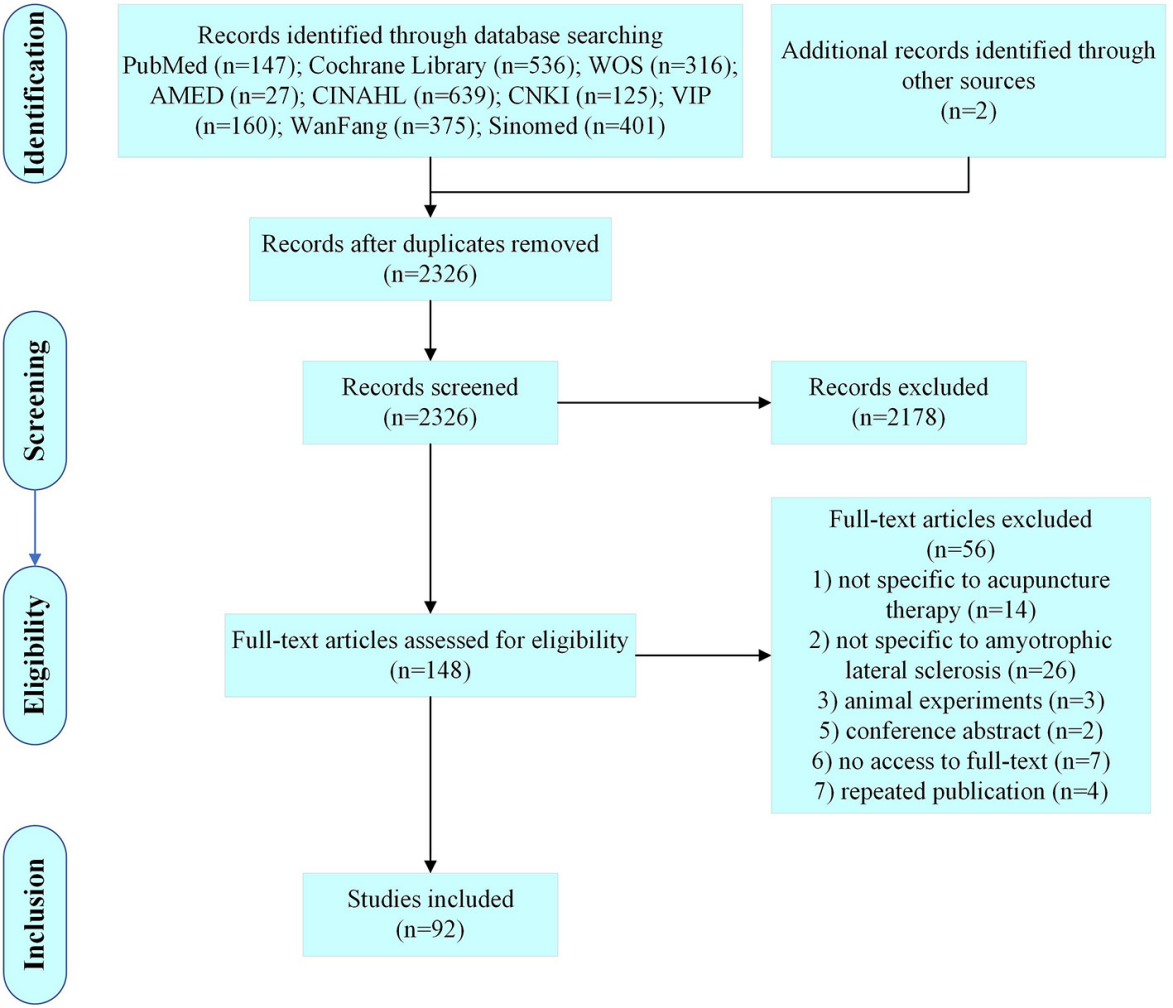
Diagram for scoping review literature identification.

A total of 2,326 articles were retrieved, and 92 articles were finally included according to the inclusion and exclusion criteria (Figure 1).

### General characteristics of the included studies

**Figure 2:**
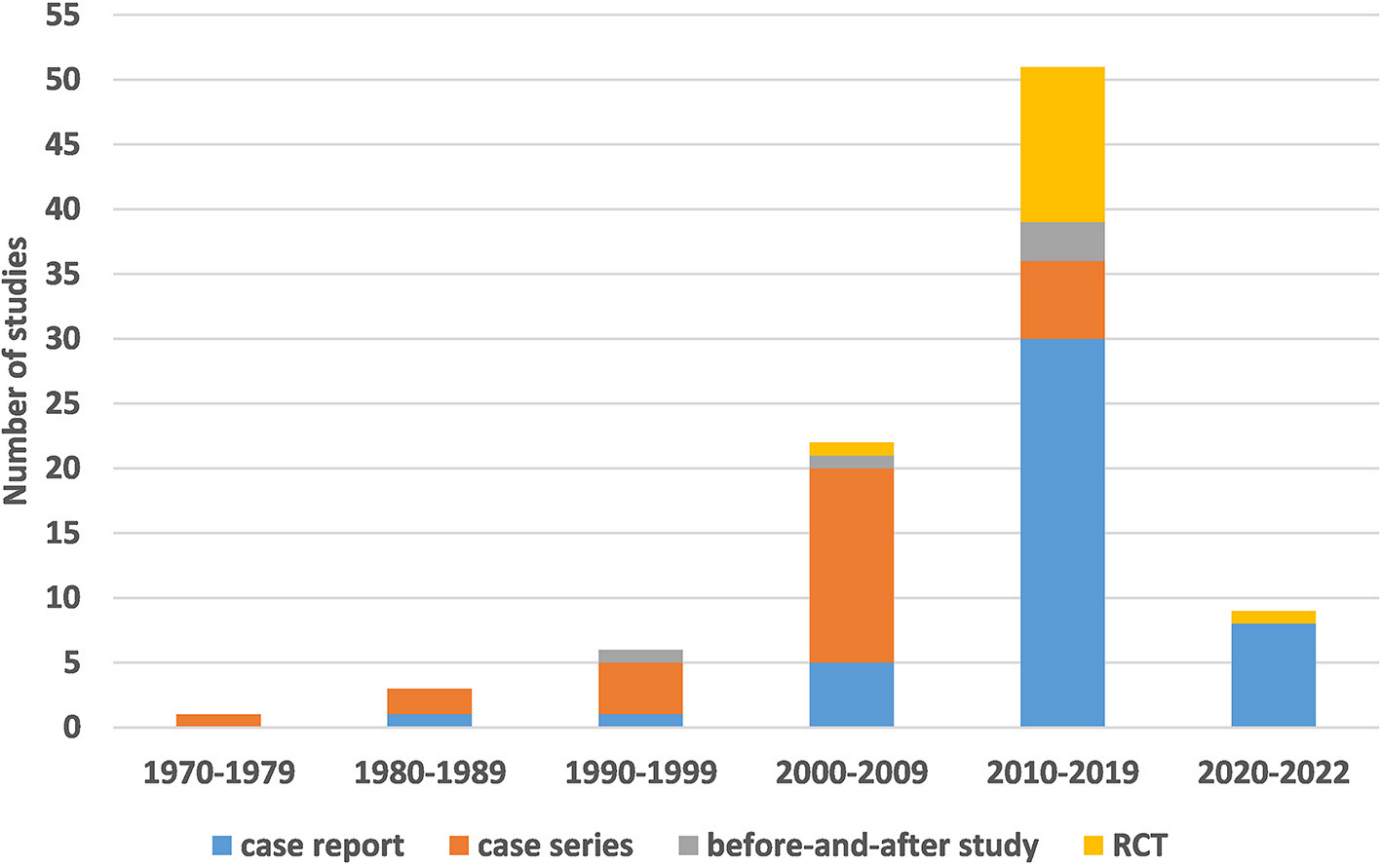
The number of different study designs used, by year of publication.

The study characteristics, including the publication year, study design, and country of origin, are shown in Table 1 and Figure 2.

**Table 1:** General characteristics of the included studies.

**Variables**	**Categories**	**Number**
Publication year	1970–1979	1
	1980–1989	3
	1990–1999	6
	2000–2009	22
	2010–2019	51
	2020–2022	9
Methodology	Case report	45
	Case series	28
	RCT	14
	Before-and-after study	5
Country	China	81
	South Korea	8
	The United States	2
	Australia	1

The earliest clinical study on acupuncture treatment for ALS was published in 1975, and the number of studies has increased in recent years. Regarding study design, the literature was divided into observational (73/92; 79.35%) and experimental (19/92; 20.65%) studies. Observational studies included case reports (45/92; 48.91%) and case series (28/92; 30.43%), while experimental studies included RCTs (14/92; 15.22%) and before-and-after studies (5/92; 5.43%). Most studies were conducted in China (81/92; 88.01%) and South Korea (8/92; 8.70%).

### Demographic characteristics of study participants

#### Number and sex of participants

A total of 1,388 patients were enrolled in 92 studies. Among them, 843 were male (843/1,388; 60.73%), 451 were female (451/1,388; 32.49%), and the sex of the patients was not determined in 5 studies, involving 94 participants (Table 2).

**Table 2:** Demographic and clinical characteristics of participants.

**Author (year); country; study design**	**Sample size, (male/female)**	**Disease type; diagnostic criteria**	**Age (y)**		**Duration of disease**	
			**Treatment group**	**Control group**	**Treatment group**	**Control group**
Li (2022); China; RCT	*n* = 50, (34/16)	ALS; the Chinese Guidelines for Diagnosis and Treatment of ALS 2012	58.14 ± 7.36	57.14 ± 8.36	11.58 ± 4.36 m	12.04 ± 3.56 m
Erik (2021); the United States; CR	*n* = 1, (1/0)	ALS; NR	60		2 y	
Sun (2021); China; CR	*n* = 1, (1/0)	ALS; NR	51		6 m	
Zhou (2021); China; CR	*n* = 1, (1/0)	ALS; NR	59		1 y	
Wu (2021); China; CR	*n* = 1, (0/1)	ALS; NR	39		24 m	
Zhang (2021); China; CR	*n* = 1, (1/0)	PBP; NR	52		6 m	
Chang (2021); China; CR	*n* = 1, (0/1)	PBP; NR	68		26 m	
Zhang (2020); China; CR	*n* = 1, (1/0)	ALS; NR	54		13 m	
Guan (2020); China; CR	*n* = 1, (1/0)	ALS; NR	58		2 y	
Zhang (2019); China; RCT	*n* = 50, (27/23)	PBP; the El Escorial Diagnostic Criteria 2000	62	61	8 m	10 m
Xu (2019); China; RCT	*n* = 40, (20/20)	ALS; NR	40.9 ± 4.1	41.4 ± 3.9	NR	
Wang (2019); China; CR	*n* = 1, (1/0)	PBP; NR	64		2 y	
Li (2019); China; RCT	*n* = 78, (46/32)	ALS; the Chinese Guidelines for Diagnosis and Treatment of ALS 2012	52.41 ± 11.48	51.08 ± 10.32	2.23 ± 0.78 y	2.31 ± 0.72 y
Sun (2018); China; CR	*n* = 1, (1/0)	PBP; NR	59		1 y	
Liu (2018); China; CR	*n* = 1, (1/0)	ALS; NR	64		2 y	
Wang (2018); China; CR	*n* = 1, (1/0)	PMA; NR	43		12 m	
Huang (2018); China; CR	*n* = 1, (1/0)	ALS; NR	61		24 m	
Xing (2018); China; CR	*n* = 1, NR	ALS; NR	53		12 m	
Wei (2018); China; before-and-after study	*n* = 17, (11/6)	ALS; the Chinese Guidelines for Diagnosis and Treatment of ALS 2012	48 ± 7		19.1 ± 5.8 m	
Liu (2017); China; CS	*n* = 20 (16/4)	ALS; NR	36.2 ± 3.6		1~5 y: 15, >6 y: 5	
Song (2017); China; CR	*n* = 1, (1/0)	ALS; NR	60		5 y	
Li (2017); China; RCT	*n* = 40, NR	ALS; Chinese Guidelines for Diagnosis of ALS (draft) 2001	38~60	39~61	NR	
Meng (2017); China; RCT	*n* = 28, (14/14)	ALS; Chinese Guidelines for Diagnosis of ALS (draft) 2001	52 ± 14	50 ± 16	20.62 ± 12.81 m	19.25 ± 11.91 m
Ahn (2017); Korea; CR	*n* = 1, (1/0)	PBP; NR	61		3 y	
Yin (2017); China; CS	*n* = 30, (18/12)	ALS: 20, PBP: 1, PMA: 6, PLS: 3; the Chinese Guidelines for Diagnosis and Treatment of ALS 2012	46.51 ± 10.29		NR	
Li (2017); China; RCT	*n* = 40, (22/18)	ALS; the Chinese Guidelines for Diagnosis of ALS (draft) 2001	42.9 ± 11.5	43.5 ± 11.8	NR	
Zhou (2017); China; CR	*n* = 1, (1/0)	ALS; NR	60		5 y	
Poovadan (2017); Australia; CR	*n* = 1, (0/1)	ALS; NR	55		4 m	
Kim (2016); Korea; CR	*n* = 1, (0/1)	ALS; NR	52		6 y	
Sun (2016); China; RCT	*n* = 80, (37/43)	ALS; NR	66.3 ± 8.2	69.1 ± 7.8	8.5~24 m	2.6~23.5 m
Zhao (2016); China; CS	*n* = 30, (20/10)	ALS: 12, PMA: 11, PLS: 1, PBP: 6; the El Escorial Diagnostic Criteria 2000	16~35: 6, 35~50: 20, >50: 4		6 m~20 y	
Wang (2016); China; CR	*n* = 1, (1/0)	PBP; NR	33		1 y	
Wu (2016); China; RCT	*n* = 56 (36/20)	ALS; NR	46.82 ± 8.96	47.62 ± 8.51	NR	
Ma (2016); China; CS	*n* = 4, (2/2)	ALS; the El Escorial Diagnostic Criteria 1994	43 ± 2.83		NR	
Pang (2015); China; CR	*n* = 1, (1/0)	ALS; NR	47		4 y	
Yuan (2015); China; CR	*n* = 1, (1/0)	ALS; NR	49		10 y	
Zhang (2014); China; before-and-after study	*n* = 32, NR	ALS: 6, PBP: 3, PMA: 17, PLS: 6; Foreign Medical Sciences Section on Neurology & Neurosurgery	20~29: 9, 30~39: 13, 40~49: 6, 50~59: 2, >60: 2		< 1 y: 7, 1~2 y: 13, 3~5 y: 3, 6~7 y: 9	
Lee (2014); Korea; CR	*n* = 1, (1/0)	ALS; NR	56		2 y	
Sun (2014); China; CR	*n* = 1, (1/0)	ALS; NR	42		2 y	
Zhang (2014); China; RCT	*n* = 60, (35/25)	ALS; NR	NR		NR	
Han (2014); China; CR	*n* = 1, (1/0)	PMA; the China Clinical Guidelines for Neurology 2006	32		1 y	
Lee (2013); South Korea; before-and-after study	*n* = 18, (14/4)	ALS; the El Escorial Diagnostic Criteria	56.06 ± 7.53		4 y	
Li (2013); China; CR	*n* = 1, (1/0)	ALS; NR	64		6 m	
Hu (2013); China; CR	*n* = 1, (0/1)	ALS; NR	38		7 y	
Lv (2013); China; CR	*n* = 1, (1/0)	PMA; NR	69		12 y	
Zhao (2013); China; CR	*n* = 1, NR	ALS; NR	65		1 y	
Lee (2013); South Korea; CR	*n* = 1, (0/1)	ALS; NR	65		2 y	
Liang (2012); China; CR	*n* = 1; (0/1)	ALS; NR	48		3 y	
Lee (2012); South Korea; CR	*n* = 1, (1/0)	ALS; NR	49		3 y	
Xie (2012); China; CR	*n* = 1, (1/0)	ALS; NR	51		6 m	
Zhou (2012); China; RCT	*n* = 29, (23/6)	ALS; the El Escorial Diagnostic Criteria 2000	76.5	71.5	NR	
Shen (2012); China; RCT	*n* = 37, (28/9)	ALS; the El Escorial Diagnostic Criteria 2000	74.5		NR	
Chen (2012); China; RCT	*n* = 30, (23/7)	ALS; the Chinese Guidelines for Diagnosis of ALS (draft) 2001	48.5	47.3	NR	
Liang (2011); the United States; CS	*n* = 2, (1/1)	ALS; NR	49, 59		12 m, 16 m	
Cui (2011); China; CR	*n* = 1, (0/1)	ALS; NR	73		18 m	
Zheng (2011); China; CR	*n* = 1, (0/1)	ALS; NR	54		2 y	
Weng (2010); China; CS	*n* = 23, (18/5)	ALS; the El Escorial Diagnostic Criteria 2000	39~68		NR	
Sun (2010); China; CR	*n* = 1, (1/0)	ALS; NR	61		2 y	
Li (2010); China; CR	*n* = 1, (1/0)	ALS; NR	54		2 y	
Shi (2010); China; CR	*n* = 1, (1/0)	PMA; NR	67		2 y	
Kim (2010); South Korea; CS	*n* = 12, (6/6)	ALS; the El Escorial Diagnostic Criteria 2000	41~50: 7, >51: 5		< 24 m: 4, 24~48 m: 5, >48 m: 3	
Ryu (2009); South Korea; CS	*n* = 2, (1/1)	ALS; NR	48, 52		1.5 y, 8 y	
Guo (2008); China; CR	*n* = 1, (1/0)	ALS; NR	40		7 m	
Ma (2007); China; CS	*n* = 20, (14/6)	ALS: 8, PBP: 2, PMA: 10; the El Escorial Diagnostic Criteria 1994	47~65		6 m~6 y	
Zhou (2006); China; CS	*n* = 20, (15/5)	ALS; NR	30~40: 7, 41~50: 9, >50: 4		NR	
Xu (2006); China; CS	*n* = 25, (23/2)	ALS; the El Escorial Diagnostic Criteria 1994	29~40: 15, >40: 10		2 d~10 y	
Pei (2006); China; CR	*n* = 1, (0/1)	PBP; NR	55		12 m	
Yuan (2005); China; RCT	*n* = 48 (28/20)	PBP; NR	44.67 ± 10.23	46.56 ± 10.31	0.87 ± 0.23 y	0.85 ± 0.21 y
Yuan (2005); China; CS	*n* = 30, (18/12)	PBP; NR	44.67 ± 10.23		0.87 ± 0.23 y	
Wang (2005); China; CS	*n* = 12, (8/4)	ALS; the Japan Diagnostic Criteria and Severity Classification of MND 1976	42~67		NR	
Peng (2005); China; CS	*n* = 30, (21/9)	PBP; NR	NR		NR	
Li (2005); China; CS	*n* = 27, (17/10)	ALS; NR	NR		NR	
You (2004); China; CR	*n* = 1, (1/0)	ALS; NR	19		2 m	
Xie (2004); China; CR	*n* = 1, (0/1)	ALS; NR	52		7 m	
Zhang (2004); China; CS	*n* = 8, (6/2)	ALS; NR	42		0.5~1.5 y	
Zheng (2004); China; CS	*n* = 20, NR	ALS; NR	NR		NR	
Zhang (2002); China; CS	*n* = 10, (3/7)	ALS; the Handbook of Diagnosis and Treatment of Neuropathy 2E000	34~49		3~18 m	
Jie (2002); China; CS	*n* = 37, (26/11)	PBP; NR	NR		NR	
Zhou (2001); China; CR	*n* = 1, (1/0)	PMA; NR	20		3 m	
Ma (2001); China; CS	*n* = 30, (19/11)	ALS; NR	15~19: 1, 20~40: 19, 41~61: 10		1~5 y: 18, 6~10 y: 10, 11~20 y: 2	
Wu (2000); China; before-and-after study	*n* = 15, (11/4)	ALS; NR	NR		NR	
Jiang (2000); China; CS	*n* = 45, (32/13)	ALS: 29, PLS: 6, PMA: 10; NR	20~29: 12, 30~39: 22, 40~49: 5, 50~59: 4, >60: 2		< 1 y: 10, 1~2 y: 14, 3~5 y: 15, 6~7 y :6	
Zhao (1999); China; before-and-after study	*n* = 30, (22/8)	PBP; NR	45~72		3~24 m	
Liang (1999); China; CS	*n* = 24, (17/7)	ALS; NR	43		5 y	
Li (1998); China; CS	*n* = 10, (8/2)	ALS; NR	43		0.5~1.5 y	
Huang (1997); China; CS	*n* = 28, (19/9)	ALS: 12, PLS: 7, PMA: 9; NR	20~35: 5, 35~50: 17, other: 6		6 m~15 y	
Zhao (1997); China; CS	*n* = 30, (22/8)	PBP; NR	45~72		3 m~2 y	
Gan (1994); China; CR	*n* = 1, (0/1)	ALS; NR	16		19 m	
Cheng (1988); China; CS	*n* = 41, (24/17)	ALS; NR	21~30: 3, 31~40: 4, 41~50: 10, 51~60: 25, >61: 4		NR	
Wu (1988); China; CS	*n* = 2, (1/1)	PMA/ALS; NR	28, 41		6 m, 8 m	
Xu (1987); China; CR	*n* = 1, (0/1)	PMA; NR	31		3 y	
Wei (1975); China; CS	*n* = 10, (7/3)	ALS; NR	36.8		NR	

RCT, randomized controlled trial; ALS, amyotrophic lateral sclerosis; CR, case report; NR, not reported; PBP, progressive bulbar palsy; PMA, progressive muscular atrophy; CS, case series.

### Disease subtype and diagnostic criteria

Among the 1,388 participants, 1,031 had ALS (1,031/13,88;74.28%), 274 had PBP (274/1,388; 19.74%), 60 had PMA (60/1,388; 4.32%) and 23 had PLS (23/1,388; 1.66%). The majority of the 92 studies did not report the diagnostic criteria applied (70/92; 76.09%). The El Escorial diagnostic criteria were most used, for a total of ten times. The 1994 version of the EI Escorial criteria was used three times (19) and the 2000 version was used six times (20). One article did not report the version in detail. The Chinese Guidelines for Diagnosis and Treatment of ALS 2012 was followed four times. The Chinese Guidelines for Diagnosis of ALS (draft) 2001 was also used four times. The China Clinical Guidelines for Neurology 2006, the Japan Diagnostic Criteria and Severity Classification of MND 1976, Foreign Medical Sciences Section on Neurology and Neurosurgery, and the Handbook of Diagnosis and Treatment of Neuropathy 2000 were each used once.

### Age and duration of disease for participants

Owing to their inconsistent description, it is difficult to accurately describe the patients' age distribution and disease duration. For example, case reports generally explicitly mentioned patients' exact age and disease duration, whereas experimental studies only described the number of patients in different age groups or the average age/average disease duration. In general, the age of the patients ranged from 16 to 76.5 years old. Six studies did not report the age profiles of their participants (6/92; 6.52%). The disease duration ranged from 2 days to 12 years, though it was not explicitly reported in 20 studies (20/92; 21.74%).

### Interventions

In the 73 observational studies, 28 types of interventions were reported, and the interventions were used 172 times in total. The maximum number of interventions used in a single study was eight and the minimum was one. Acupuncture treatments included body acupuncture (62/73; 84.93%), acupoint injection (13/73; 17.81), electroacupuncture (10/73; 13.70%), scalp acupuncture (3/73; 4.11%), acupoint massage (3/73; 4.11%), pharmacopuncture (2/73; 2.74%), Sa-am acupuncture (2/73; 2.74%), needle-embedding therapy (2/73; 2.74%), auricular acupuncture (1/73; 1.37%), eye acupuncture (1/73; 1.37%), venom pharmacopuncture therapy (1/73; 1.37%), fire needle therapy (1/73; 1.37%), and plum blossom needling (1/73; 1.37%). A brief introduction of included acupuncture treatments is shown in Supplementary File 2. Other interventions included herbal decoction (31/73;42.47%), moxibustion (9/73; 12.33%), oral Western medicine (7/73; 9.59%), intravenous injection (7/73; 9.59%), cupping (6/73; 8.22%), bloodletting (3/73; 4.11%), intravenous injection (3/73; 4.11%), massage (3/73; 4.11%), dysphagia rehabilitation and electrical stimulation (1/73; 1.37%), speech therapy (1/73; 1.37%), nutritional support (1/73; 1.37%), non-invasive BIPAP ventilator (1/73; 1.37%), deglutition training (1/73; 1.37%), intramuscular injection (1/73; 1.37%), physical treatment (1/73; 1.37%), and exercise (1/73; 1.37%).

In 19 experimental studies, 15 different types of interventions were reported in the treatment group, and the interventions were used 46 times in total. Each study used at least one intervention. Acupuncture treatments included body acupuncture (10/19; 52.63%), acupoint massage (3/19; 15.79%), acupoint paste (1/19; 5.26%), electroacupuncture (1/19; 5.26%), needle warming through moxibustion (1/19; 5.26%), Sa-am acupuncture (1/19; 5.26%), acupoint injection (1/19; 5.26%), and scalp acupuncture (1/19; 5.26%). Other interventions included oral Western medicine (9/19; 47.37%), herbal decoction (9/19; 47.37%), moxibustion (3/19; 15.79%), intravenous injection (2/19; 10.53%), dysphagia therapeutic apparatus (1/19; 5.26%), stellate ganglion block (1/19; 5.26%), bloodletting (1/19; 5.26%), and swallowing nerve and muscle electrical stimulator (1/19; 5.26%). Six interventions were reported in the control group: oral Western medicine, acupuncture, herbal decoction, swallowing nerve and muscle electrical stimulator, dysphagia therapeutic apparatus, and intravenous injection.

Of all the included studies, ten did not clearly report the frequency of acupuncture treatment. Among the other 82 studies, the frequency of acupuncture treatment ranged from once each month to three times per day, with the most chosen frequency being once per day (49/92; 53.26%). Three articles reported that the frequency of acupuncture treatment was adjusted according to the course of treatment and condition of the patient. Ten studies did not clearly report the duration of acupuncture treatment, and 11 articles only reported the approximate interval. In the other 71 articles, the duration of acupuncture treatment ranged from 5 days to 3 years. The most frequently selected treatment duration was 1–4 weeks (23/92; 25.00%). The second was 3 months (10/92; 10.87%). The third were 5–8 weeks (8/92; 8.70%) and 2 months (8/92; 8.70%).

### Acupoints

According to the international standard of acupoints, 201 acupoints with clear names and positions were included, including 131 meridian acupoints, 24 extra acupoints, five acupoint areas covered by scalp acupuncture, four acupoints covered by eye acupuncture, and four acupoints covered by auricular acupuncture. The 22 acupoints with the highest frequency of use are shown in Table 3.

**Table 3:** The 22 acupoints with the highest frequency of use.

**Acupoint**	**Number**	**Acupoint**	**Number**
ST36	57	PC6	27
LI4	53	BL20	26
LI11	43	GV14	26
SP6	39	CV23	25
GB20	38	BL18	20
GV20	31	CV12	20
BL23	28	CV6	20
EX-B2	28	SP9	19
GB34	28	CV4	18
LI10	27	KI3	18
LI15	27	LR3	17

### Outcomes

A total of 48 types of outcomes were reported in all studies and used 206 times in total. A single study always included at least one outcome and had 11 outcomes at the most. The most common outcome was clinical symptoms (64/92; 69.57%). Other outcomes with a high frequency of use included effective rate (35/92; 38.04%), muscle strength (28/92; 30.43%), Amyotrophic Lateral Sclerosis Functional Rating Scale (ALSFRS)/Amyotrophic Lateral Sclerosis Functional Rating Scale-Revised (ALSFRS-R) (7/92; 7.61%), and water-swallowing test (7/92; 7.61%).

### Adverse events

Most studies did not report any adverse events (86/92; 93.48%). No severe adverse events were observed in four of the studies (4/92; 4.35%). A case series in 2011 reported one case of fatigue, muscle aches, light headedness, increased edema, and complaints of feeling feverish after acupoint injection (21). An RCT in 2012 reported two cases of diarrhea in the treatment group after acupoint massage, acupoint injection, and oral medicine.

### Significant findings

In 73 observational studies, 42 reported improvements in symptoms, such as dysphagia, sialorrhea, fatigue, appetite, muscle wasting, dyspnea, constipation, slurred speech, pain, sweating, insomnia, fasciculations, shortness of breath, sleep, and anorexia in patients with ALS. Twenty studies reported the effective rate. Six studies reported significant improvements in ALSFRS, ALSFRS-R, Karnofsky Performance Status (KPS), electromyogram (EMG) reading, numerical rating scale (NRS), or Pittsburgh Sleep Quality Index (PSQI) scores. Lee reported improvements in end-tidal carbon dioxide, SpO_2_, respiratory rate, and pulse in a patient after using Sa-am acupuncture with different manipulations (22). One study reported a PBP patient had improvement in speech mechanism screening test (SMST) and speech handicap index (SHI) after acupuncture and some other Korean medicine treatments. One study reported that acupuncture could help maintain SpO_2_, end-tidal carbon dioxide, and expiratory tidal volume (Vte) in ALS patients with respiratory failure. One study reported that scores from the 40-item Amyotrophic Lateral Sclerosis Assessment Questionnaire (ALSAQ-40), Modified Norris Scale and Appel Scale did not improve significantly after treatment. One study reported no statistical significance in the mean ALSFRS-R or Medical Research Council Scale (MRC) scores after intervention (23). In a case series reported by Ryu, symptoms were relieved, while there was no improvement in the Korean version of the Amyotrophic Lateral Sclerosis Functional Rating Scale-Revised (K-ALSFRS-R) or Amyotrophic Lateral Sclerosis Severity Scale (ALSSS) score after acupuncture, moxibustion, cupping, herbal decoction, oral Western medicine, and physical treatment, demonstrating that oriental medical treatment is effective for local symptoms of ALS but brings no functional improvement (24). All interventional studies reported positive results, and 15 studies reported the effective rate. See Tables 4, 5 for further details.

**Table 4:** Summary of included observational studies.

**Author (year); study design**	**Intervention, frequency; duration**	**Acupoints**	**Outcomes**	**Significant findings**
Erik (2021); CR	Scalp acupuncture, auricular acupuncture; several years	Upper 1/5th motor region, Chorea/Tremor area, Praxis area, Foot motor sensory area, Mouth, Trachea, Shenmen, Point zero	ALSFRS	ALSFRS improved 1 point for 1~4 days with each treatment; stable symptoms for few years
Sun (2021); CR	Acupuncture 6/w, herbal decoction 2/d; 12 w	EX-B2, GV20, LI15, TE14, SI9, LI11, LI10, TE5, LI4, ST31, ST32, ST34, ST36, GB34, SP6, LR3, KI3	Clinical symptoms	Improved fasciculation, dysphagia, and fatigue
Zhou (2021); CR	Acupuncture 1/d; 1 m	PC6, GV26, GB20, GB12, BL10, EX-B2, CV6, SP10, GB34, LR3, HT1, LU5, BL40, SP6	Clinical symptoms, muscle strength	Decreased muscle tension, improved muscle strength
Wu (2021); CR	Acupuncture 1/d, electroacupuncture 1/d, Buzhongyiqi decoction 2/d; 3 m	CV12, CV6, CV4, LI15, LI11, LI4, ST34, ST36, SP9, SP6, SP10	Clinical symptoms, muscle strength	Improved dysphagia, fatigue, and muscle strength
Zhang (2021); CR	Acupuncture, electroacupuncture, bloodletting 5/w; 4 w	SP3, CV23, Waijinjin Yuye, GV20, ST36, SP6, ST40, GB20, TE17, EX-B2, EX-HN12, EX-HN13	Clinical symptoms	Improved dysphagia and dysphonia
Chang (2021); CR	Acupuncture 3/w, herbal decoction 2/d; 3 y	GB20, Gongxue, Tunyan, Fayin, Zhiqiang, Waijinjin Yuye, CV23, LI4, GB15, GV24, CV20	Clinical symptoms	Improved dysphagia and sialorrhea
Zhang (2020); CR	Acupuncture 2/w, panlong moxibustion 2/w; 2 m	PC6, SP4, CV12, CV9, BL21	Clinical symptoms, muscle strength	Improved fatigue, appetite, and muscle strength
Guan (2020); CR	Acupuncture 1/d, acupoint injection 1/d, intravenous injection 1/d; 4 w	EX-B2, BL18, BL20, BL21, BL23, BL54, BL40, BL60, LI11, LI4, CV6, CV4, ST36, GB34, ST34, ST40, SP6, KI3, LR3	Clinical symptoms, muscle strength	Improved fatigue, muscle strength, and muscle tension
Wang (2019); CR	Acupuncture 5/w; 6 w	GB20, GV16, GV20, CV23, CV24, PC6, LI4, ST36, ST40, LR3, SP6, CV6, CV12, lower 2/5 motor region	Clinical symptoms	Improved dysphagia, dysphonia, and sialorrhea
Sun (2018); CR	Acupuncture 6/w, electroacupuncture 6/w; 2 m	CV23, Waijinjin Yuye, GV20, EX-HN3, ST9, LI4, ST36, SP6, ST40, KI3, LR3, GB20, BL10, Gongxue, EX-B2	Clinical symptoms	Improved dysphagia, dysphonia, sialorrhea, and weight 3 kg↑
Liu (2018); CR	Acupuncture 1/d, herbal decoction 2/d; 2 m	LI15, TE14, SI9, LI11, LI10, TE5, LI4, CV23, ST25, CV12, CV6, CV4, ST36, SP9, ST38, SP6, EX-B2, GV20, GV14, BL15, BL17, BL18, BL20, BL21, BL23, BL26, KI3	Clinical symptoms	Improved muscle weakness
Wang (2018); CR	Acupuncture, 1/d; 9 w	GV20, GV29, GB7, LI15, LI11, LI10, LI4, CV12, CV6, CV4, ST36, SP10	Clinical symptoms	Improved fatigue
Huang (2018); CR	Acupuncture 1/d, cupping 1/d; 2 m	LI11, LI15, LI14, LI10, LI4, GB34, ST36, SP6, TE10, TE5, SI11, ST30, ST32, BL18, BL15, BL20, BL13, BL23, BL17, CV12, CV10, CV6, CV4, ST24, ST26, upper rheumatic point	Clinical symptoms	Improved fatigue and fasciculation
Xing (2018); CR	Acupuncture 6/w, oral Western medicine; 2 m	LI11, LI4, CV12, ST36, SP9, ST40, SP6, SP10, SP8, GB39, ST41, BL13, BL15, BL18, BL20, BL23, BL54, GV3, GV4, GV9, GV14, GV12, GV8, GV5	Clinical symptoms, muscle strength	Improved fatigue, appetite, and muscle strength
Liu (2017); CS	Acupuncture 1/d, acupoint injection 1/2d, herbal decoction 2/d; 1 m	GV14, EX-B2, GB34, GV4, CV4, CV6, BL20, BL21, BL23, LI15, TE14, SI9, LI11, LI10, PC6, LI5, LU10, ST34, ST36, ST41, BL40, GB30, LI4, SP6, SP10, LR3	Clinical symptoms, muscle strength, ER	Markedly effective: 10; effective: 6; total ER: 80.00%
Song (2017); CR	Acupuncture 2/d, electroacupuncture 1/d, cupping 1/d, moxibustion 1/d, herbal decoction 2/d; 2 w	PC6, GV26, HT1, LI15, LI4, EX-UE9, EX-B2, GB20, GB12, BL10	Clinical symptoms	Improved muscle wasting
Ahn (2017); CR	electroacupuncture 1/d, acupuncture 1/d, pharmacopuncture 1/d, cupping 1/d, herbal medicine 3/d, dysphagia rehabilitation and electrical stimulation 1/d, speech therapy 1/d, oral Western medicine; 25 d	GV26, CV24, CV23, GV26, GB20, GV16, GB21, GV14	SMST, tongue and orbicularis oris motility, SHI	SMST and tongue and orbicularis oris motility showed tendency for improvement; SHI: speech domain 1 point↑, psycho-social domain 1 point ↓, other domain 2 point ↑
Yin (2017); CS	Acupuncture, herbal decoction; NR	LI11, LI4, EX-B2, ST31, GB31, ST36, GB34, SP6, BL20, BL21, CV12, BL18, BL23, SP10, BL40	muscle strength, ER	Markedly effective: 10; effective: 15; total ER: 83.33%
Zhou (2017); CR	Acupuncture 1/d; 2 w	PC6, GV26, GB20, GB12, BL10, EX-B2, SP10, GB34, LI4, HT1, LI11, SP6, GV14, BL18, BL23, GB39, ST36	Clinical symptoms, muscle strength	Improved muscle weakness and muscle wasting
Poovadan (2017); CR	Acupuncture, electroacupuncture (2/w for 8 w, 1/m for 6 m), riluzole 50mg 2/d, nutritional support; 3 y	LI15, LI11, LI5, LI4, LI10, SI9, LU5, TE5, GB30, GB31, ST31, ST32, ST36, SP6, GB39, ST41, BL32, BL40, BL57, GB40, GB41, KI3, SP9, BL20, BL21, CV12, LR13, SP6, BL18, BL23, LR8, GB34, CV4, GV4	Clinical symptoms, KPS, muscle strength	KPS: 70 → 100, symptoms free, normal muscle strength, and reduction in muscle wasting
Kim (2016); CR	Ogapijangchuk-tang and acupuncture; 12 d	LU8, KI7, SP3, KI3, HT3, GB34, KI10, LR8, LU8, LR4, HT8, LU10, LU5, ST36	K-ALSFRS-R, GAS	K-ALSFRS-R: 30 → 26, significant improvement in GAS, and longer walking distance
Zhao (2016); CS	Acupuncture, acupoint injection, moxibustion 3/w, herbal decoction 2/d; 3 m	GB21, SI11, LI15, LI11, TE5, LI4, TE3, GB30, ST31, GB31, ST32, BL40, GB34, ST36, BL57, BL60, ST41, GB41, GB20, TE17, GV15, CV23, EX-HN12, EX-HN13, CV12, ST40, SP9, SP6, PC6	Clinical symptoms, ER	Markedly effective: 2; effective: 20; total ER: 73.30%
Wang (2016); CR	Acupuncture 5/w, bloodletting 5/w; 4 w	EX-HN12, EX-HN13, GB20, GB12, TE17, CV24, EX-B2, LI4, ST36, SP9, ST40	Water-swallowing test, clinical symptoms	Water-swallowing test level 4 → 1, improved dysphagia, dysphonia, tongue fasciculation and sialorrhea; weight 4 kg ↑
Ma (2016); CS	Acupuncture, 1/d, 10d/course, 2 days rest between courses; 3 courses	LI15, LI4, LI11, ST32, ST36, ST41	ALSFRS-R, ALSAQ-40, modified Norris scale, Appel Scale, EMG, NCV	ALSFRS-R: 31.70 ± 3.40 → 30.50 ± 3.87 (*P* < 0.05); ALSAQ-40: 124.30 ± 23.64 → 123.00 ± 22.42 (*P* > 0.05); modified Norris scale: 101.80 ± 13.27 → 100.80 ± 12.12 (*P* > 0.05); Appel scale: 79.80 ± 4.35 → 79.00 ± 5.29 (*P* > 0.05); significant difference in denervated potential in sternocleidomastoid muscle and paraspinal muscle (*P* < 0.05); no adverse events
Pang (2015); CR	Acupuncture 6/w, electroacupuncture 6/w; 4 w	ST44, SP2, KI2, LI4, BL21, BL20, BL23, GV20, EX-B2	Clinical symptoms	Improved muscle weakness
Yuan (2015); CR	Acupuncture 1/d; 6 m	LI4, LI5, LI6, LI7, LI10, LI11, LI13, ST31, ST32, ST34, ST35, ST36, ST38;SP10, SP9, SP6, LR3, GB41;BL5, BL6, BL7, BL8;GV24, ST8, GB20, ST25, SP15, CV12, CV10	Clinical symptoms, muscle strength	Improved muscle weakness
Lee (2014); CR	Non-invasive BIPAP ventilator, acupuncture, pharmacopuncture, herbal medicine; 31 m	LI4, LI10, LI11, ST34, ST36, GB39, GB40, ST35, CV12, GB20, GV16, GV15, GV14, GB21, BL23, BL52, SP11, LR13	SpO_2_, EtCO_2_, Vte	The SpO_2_, EtCO_2_ of patient maintained in normal range for 2 years and 7 months. The Vte did not worse. Respiration management with Korean medical treatment and non-invasive BIPAP ventilator could be effective in ALS patient with respiratory failure.
Sun (2014); CR	Electroacupuncture 1/d, herbal decoction 2/d; 8 w	GV20, GB7, EX-HN1, GB6, GV24, GV29, EX-B2	Clinical symptoms, muscle strength	Improved fatigue and muscle strength, weight: 3kg↑
Han (2014); CR	Acupuncture 1/2d; 16 w	EX-B2, EX-UE9, CV4, ST36, LI15, Jianneiling, Jianwailing, Jianqian, Taijian, LI11, LI4, TE3, LI10, TE5	Clinical symptoms, muscle strength	Improved fatigue and muscle strength
Li (2013); CR	Acupuncture 2/d, deglutition training 2/d; 6 w	GB20, EX-HN14, Zhiqiang, Gongxue, Tunyan, Fayin, Zhifanliu, CV23, Waijinjin Yuye	Water-swallowing test, clinical symptoms	Water-swallowing test level 5 → 1, improved dysphagia
Hu (2013); CR	Electroacupuncture 1/d, acupoint injection 1/d, Shenlingbaizhu decoction 2/d; 1 m	LI15, LI11, LI10, LI13, LI4, ST36, ST31, ST32, ST37, SP6, SP9, GB34, LR3, KI3	Clinical symptoms, muscle strength	Improved fasciculation and muscle strength
Lv (2013); CR	Acupuncture 1/2d; 12 w	PC6, GV26, HT1, LU5, GB20, EX-B2, GV20, GV29, GV24, GB21, SI11, LI15, LI14, LI11, LI10, TE5, LI4	Clinical symptoms	Improved muscle weakness and muscle wasting
Zhao (2013); CR	Acupuncture 1/d, herbal decoction 2/d; 2 w	EX-B2, BL17, BL18, BL47, LR14, CV17, CV12, CV6, LU5, LU7, GV13, BL17, BL18, BL20, BL23, ST36, SP6, CV6	Clinical symptoms	Improved fatigue, muscle strength and dyspnea
Lee (2013); CR	Acupuncture 1/d, Ecklonia Cava Extract 500mg 1/d; 28 w	HT7, SP6, GB12, GB20	NRS, PSQI	sleep time 2 → 7 h; NRS↓, PSQI 12 → 8; this treatment may be effective in improving sleep quality
Liang (2012); CR	Acupoint massage 1/d; 3 m	GV20, GB20, GB21, LI11, LI4	Clinical symptoms, muscle strength	Improved muscle weakness
Lee (2012); CR	Sa-am acupuncture 3/d; 5 d	SP3, LU9, HT8, LU10, HT9, LR1, SI8, KI10, KI10, LR8, LU8, LR4	EtCO_2_, SpO_2_, RR, pulse	EtCO_2_ decreased more after lung tonification treatment; pulse decreased more after heart tonification treatment; RR decreased more after liver tonification treatment; SpO_2_ increased more after liver tonification treatment
Xie (2012); CR	Acupuncture 1/d, herbal decoction 2/d; 4 w	LI11, LI10, LU10, PC6, LI4, ST36, SP10, ST34, SP9, GB34, ST40, SP6, ST42, KI3	Clinical symptoms	Improved muscle strength, dyspnea, and constipation; successful ventilator weaning
Liang (2011); CS	Acupoint injection 5/w, intramuscular injections, oral Western medicine; 4 w	GV20, EX-B2, GV14, LI11, TE5, GB34, ST36, ST4, ST6, ST21, ST25, ST29, BL17, LR14, BL20, BL21, LI4, LI15, SI6, GB30, ST37, ST41, BL60	Clinical symptoms, muscle strength	Improved speech, swallowing, and muscle strength; ongoing therapy may be necessary in order to maintain these positive effects; one case of fatigue, muscle aches, headedness, increased edema, and complaints of feeling feverish
Cui (2011); CR	Acupuncture, 1/d; 1 m	BL10, GB12, GB20, CV23, Panglianquan	Clinical symptoms	Improved dysphagia
Zheng (2011); CR	Acupuncture 1/d; 8 w	EX-HN12, EX-HN13, GB20, GV26, HT1, LU5, PC6, BL40, SP6, BL18, BL23, LI4, ST36, GB34, KI3	Clinical symptoms, muscle strength	Improved muscle strength, fatigue, dysphagia, and slurred speech
Weng (2010); CS	Acupuncture 1/2d, moxibustion 1/2d, massage 1/2d, herbal decoction 2/d; 60~90 d	LI11, TE5, LI4, GB20, LI10, PC6, ST36, ST37, ST39, SP6, ST44, GB34, BL20, BL23, BL18, GV4, CV17, GV14, CV8, CV4, CV6	Clinical symptoms, ER	Cured: 9; markedly effective: 2; effective: 7; total ER: 78.26%
Sun (2010); CR	Eye acupuncture 1/d, 15d/course; 4 courses	Spleen region, kidney region, upper warmer region, middle warmer region	Clinical symptoms, muscle strength	Improved muscle weakness and muscle wasting
Li (2010); CR	Acupuncture 1/d; 16 w	EX-B2, LI-meridian, ST-meridian	Clinical symptoms, muscle strength	Improved muscle strength, dysphagia, and slurred speech
Shi (2010); CR	Acupuncture 2/d, herbal decoction; NR	PC6, GV26, SP6, GV20, GV23, EX-HN3, HT1, BL40, EX-B2	Clinical symptoms, muscle strength	Improved muscle weakness and fasciculation
Kim (2010); CS	Sa-am acupuncture, needle-embedding therapy, bee venom pharmacopuncture therapy 1/d, herb decoction; 3 m	ST36, SP9, LR3, CV23, CV17, GV1, LI11, SP6, GV16, GV14	ALSFRS-R, MRC	Mean ALSFRS-R: 1 m: 29.08 ± 7.99, 2 m:28.70 ± 7.17, 3 m: 28.16 ± 8.23, 1 y: 21.33 ± 9.93; mean MRC: 1 m:25.34 ± 8.45, 2 m: 25.34 ± 8.45, 3 m: 21.56 ± 9.20 (not statistically significant)
Ryu (2009); CS	Acupuncture, moxibustion, cupping, herbal decoction, physical treatment, oral Western medicine; 4~16 w	LI4, LR3, ST36, CV12, CV24, SP9, KI3, BL9, GV17, SI3, TE3, GB21, LI11, GB39, LI10, LI15, Jianneiling, SI3, LI14, SI14, SI11, BL10, BL25, BL60, GB30, GB34, Nie three needles, CV22, CV23, TE14, SI9	K-ALSFRS-R, ALSSS, VAS	Improved cervical and shoulder pain, knee pain, cold sweating, and insomnia; no improvement in K-ALSFRS-R or ALSSS; oriental medical treatment is only effective on local symptoms of ALS
Guo (2008); CR	Acupuncture 2/d, massage, cupping, herbal decoction, oral Western medicine; 50 d	LI15, LI14, LI13, LI11, LI10, LI5, LI4, ST31, ST34, ST35, ST36, ST37, ST39, ST40, ST41, EX-B2	Clinical symptoms, muscle strength	Improved muscle strength, muscle waste, and fasciculation
Ma (2007); CS	Acupuncture 1/d, herbal decoction 2/d; 1~2 m	LI15, TE14, SI9, SI11, LI11, TE5, LI4, GB30, GB34, SP3, ST36, ST42, LR3, KI3, CV23, Waijinjin Yuye, CV22, GV15, CV12, ST40, SP9, PC6, LU10, LI2, LI3, SI2, SI3	Clinical symptoms	Improved dysphagia, sialorrhea, and muscle weakness
Zhou (2006); CS	Acupuncture 1~2/d; 12~48 w	GV16, EX-B2	Clinical symptoms, ER	Markedly effective: 60.00%, effective: 25.00%, ineffective: 15.00%
Xu (2006); CS	Acupuncture 1/d; 36 d	GV14, BL13, BL21, BL18, BL23, GB21, LI15, LI11, LI4, LI5, ST31, ST34, ST36, ST41	EMG, muscle strength, ER	Cured: 6; markedly effective: 12; effective: 5; total ER: 92.00%
Pei (2006); CR	Acupuncture 1/d; 4 w	GB20, LI11, LI10, LI4, ST36, SP6, KI3, LR3	Clinical symptoms, water-swallowing test	Improved dysphagia and fatigue, water-swallowing test 4 → 2
Yuan (2005); CS	Acupuncture 2/d, Jiweiling injection 1/d; 12 w	PC6, GV26, GV23, GV20, GV29, GB20, TE17, CV23	ER	Markedly effective: 26.70%; effective: 56.60%; ineffective: 16.70%; total ER: 83.30%
Wang (2005); CS	Acupuncture 1/d, moxibustion 2~3/m, acupoint injection 3/w, herbal decoction, intravenous injection; 4~12 w	GB21, SI11, LI15, LI11, TE5, LI4, TE3, GB30, ST31, GB31, ST32, BL40, GB34, ST36, BL57, BL60, ST41, GB41, GB20, TE17, GV15, CV23, EX-HN12, EX-HN13, CV12, ST40, SP9, SP6, PC6	Clinical symptoms	Improved muscle weakness, dysphagia, and sialorrhea
Peng (2005); CS	Acupuncture 1/d; 10 d	CV23, GB20, LI4, ST36	Clinical symptoms, ER	Cured: 27; remarkedly effective: 2; ineffective: 1; total ER: 97.00%
Li (2005); CS	Acupuncture 1/d; acupoint injection 1/2d, Chinese herbal decoction 2/d; 60~100 d	LI15, LI11, LI10, LI4, LI3, ST31, ST34, ST36, GB34, GB39, ST41, ST43, CV23, Shanglianquan, BL20, BL21, BL18, BL23	Clinical symptoms, ER	Cured: 18.5%; markedly effective: 40.70%; effective: 25.90%; total ER: 85.20%
You (2004); CR	Acupuncture 1/d, acupoint injection 1/d; 2 m	LI4, LI11, LI15, SI9, GB30, GB31, GB34, SP6, SP9, LU5, Eshi	Muscle strength	Improved muscle strength and muscle wasting
Xie (2004); CR	Acupuncture, moxibustion, herbal decoction; NR	GV20, EX-HN1, GB20, GV16, GV14, LI11, LU7, LI4, BL13, BL17, GV9, BL43, KI1, SP9, ST40, CV22, BL17	Clinical symptoms	Improved dysphonia and muscle weakness
Zhang (2004); CS	Acupuncture 1/d, herbal decoction 2/d; 2 m	EX-B2, BL18, BL23, BL20, SP6, KI3, ST36, SP10, GB34	Clinical symptoms, EMG, ER	Cured: 1; markedly effective: 3; effective: 2; total ER: 75.00%
Zheng (2004); CS	Acupuncture 1/d, acupoint injection 1/d, needle-embedding therapy 1/m; NR	GV14, LI10, LI11, LI4, ST36, GB34, GB39, GV4, CV6, SP10, LR3, BL20, CV4, PC6, LU10	Clinical symptoms, ER	Cured: 4; markedly effective: 10; effective: 4; total ER: 90.00%
Zhang (2002); CS	Acupuncture 1/d, moxibustion 1/d; 2 w	GV-meridian, GV20, GB20, BL20, BL21, EX-B2, LI4, LI11, ST36, ST40	Clinical symptoms, ER	Markedly effective: 30.00%; effective: 40.00%; total ER: 70.00%
Jie (2002); CS	Acupuncture 2/d, scalp acupuncture 2/d; 3 m	PC6, GV26, SP6, GV23, GV20, GV29, GB20, GB12, BL10, TE17, CV23, lower 2/5th motor region	Clinical symptoms, ER	Cured: 4; markedly effective: 12; effective: 16; total ER: 83.00%
Zhou (2001); CR	Acupuncture 1/d, electroacupuncture 1/d, acupoint injection 1/d; 1 m	LI15, LI14, LI10, LI11, LI4, TE5, LU7, SI3, ST36, GV20, EX-UE9, EX-B2, BL20, BL21, BL18, BL23, BL13	Clinical symptoms, muscle strength	Improved muscle wasting and muscle strength
Ma (2001); CS	Acupuncture 1/d, acupoint injection 1/d; NR	LI15, LI11, LI10, LI5, GV14, ST31, ST34, ST36, ST41, GV3	Clinical symptoms, muscle strength, ER	Total ER: 63.30%
Jiang (2000); CS	Acupuncture 1/d, herbal decoction 2/d; 3 m	ST36, ST31, ST32, ST41, LI15, LI11, LI4, LI10, SP9, BL20, BL18, BL23, GV14, GV12, EX-B2, CV6, BL21, CV4, GV4, KI3, SP6, GB39, ST40, ST44, BL17, GB20, GV16, CV23	Clinical symptoms, muscle strength, ER	Markedly effective: 15; effective: 22; total ER: 82.22%
Liang (1999); CS	Acupoint injection 1/2d, herbal decoction 2/d; NR	GV14, PC6, ST36	Clinical symptoms, EMG	Cured: 2; markedly effective: 9; effective: 11
Li (1998); CS	Acupuncture 5/w, acupoint injection 1/2d, massage 5/w, herbal decotion 1/d; NR	LI15, LI11, LI4, ST31, ST34, ST36, BL18, BL23, GB39, GB34	Clinical symptoms, EMG, ER	Cured: 1; markedly effective: 3; effective: 3; total ER: 70.00%
Huang (1997); CS	Fire needle therapy 1/3d, acupoint massage 1/d, herbal decoction 2/d, cupping 1/5d, moxibustion 1/3d, bloodletting 1/15d; 2.5 m~9 m	CV4, GV14, CV8, BL18, BL23, LI10, ST36, GV20, PC6, LI4, BL57, SP6, EX-UE10	Clinical symptoms, ER	Cured: 12; markedly effective: 8; effective: 2; total ER: 92.80%
Zhao (1997); CS	Acupuncture 2/d, scalp acupuncture 2/d; 3 m	PC6, GV26, GV23, GV20, EX-HN3, GB20, GB12, BL10, TE17, CV23, lower 2/5 motor region	Clinical symptoms, ER	Cured: 3; markedly effective: 9; effective: 13; total ER: 83.00%
Gan (1994); CR	Acupuncture 1/d, acupoint massage 1/d; 6 w	Upper 1/3 motor region, GB31, GB34, ST36, GB39, BL60, ST32, SP9, SP6, ST41, BL20, BL18, BL17, BL15, GV14, SI11, GV16, GV20, HT7, PC7, LU9, PC6, HT3, LU5, LU1	Clinical symptoms, muscle strength	Improved fasciculation, fatigue and muscle strength
Cheng (1998); CS	Acupuncture 1/1~2d, herbal decoction 2/d, exercise; NR	GV16, GV14, EX-B2, Eshi	Clinical symptoms, survival time, viability	cured: 6; markedly effective: 11; effective: 24
Wu (1988); CS	Acupuncture, plum blossom needling 1/d for 1 m, then 1/2d, 20 times a course; 5 courses	GV20, BL20, BL23, GV4, KI3, CV17, CV4, CV6, SP10, SP6, GV14, LI11, TE5, LI4, ST36, GB20, LI10, PC6, SP9, LR3	Clinical symptoms	Improved fatigue and muscle waste
Xu (1987); CR	Acupuncture 1/d, herbal decoction 2/d; 20 w	LI15, LI10, TE4, LI4, BL23, GB30, GB34, ST41, LI13, TE5, TE3, GB31, GB39, GB40, ST36	Clinical symptoms, muscle strength	Improved muscle strength
Wei (1975); CS	Acupuncture 1/d; 10 d	GV14, LI15, LI11, LI10, LI4, LU10, LU9, ST36, ST32, GB31, GB30, GB34, GB39	NR	cured: 5

CR, case report; ALSFRS, Amyotrophic Lateral Sclerosis Functional Rating Scale; ER, effective rate; SMST, speech mechanism screening test; SHI, speech handicap index; NR, not reported; KPS, Karnofsky Performance Status; CS, case series; K-ALSFRS-R, Korean version of the Amyotrophic Lateral Sclerosis Functional Rating Scale-Revised; GAS, Global Assessment Scale; ALSFRS-R, Amyotrophic Lateral Sclerosis Functional Rating Scale-Revised; ALSAQ-40, 40-item Amyotrophic Lateral Sclerosis Assessment Questionnaire; EMG, electromyogram; NCV, nerve conduction velocity; BIPAP, bilevel positive airway pressure; SpO_2_, peripheral oxygen saturation; EtCO_2_, end-tidal carbon dioxide; Vte, expiratory tidal volume; NRS, numerical rating scale; PSQI, Pittsburgh Sleep Quality Index; RR, respiratory rate; MRC, Medical Research Council Scale; ALSSS, Amyotrophic Lateral Sclerosis Severity Scale; VAS, Visual Analog Scale.

**Table 5:** Summary of included interventional studies.

**Author (year); study design**	**Intervention, frequency; duration**		**Acupoints**	**Outcomes**	**Significant findings**
	**Treatment group**	**Control group**			
Li (2022); RCT	Acupoint paste 1/d, Jianpiyifei decoction 1/d, oral Western medicine; 2 w	Oral Western medicine; 2 w	BL43, ST36, GV13, BL20, SP6	TCM syndrome score, ER, PaO_2_, PaCO_2_, ALSFRS	Improved shortness of breath, dyspnea, fatigue, anorexia, sweating, PaO_2_, and score of limb symptom and total scores of ALSFRS in treatment group. The total ER in treatment group and control group were 88.46% and 62.50% (P < 0.05)
Zhang (2019); RCT	Shallow needling 1/d, Shenqiqiangli capsule 3/d; 20 d	Acupuncture 1/d, Shenqiqiangli capsule 3/d; 20 d	EX-HN14, GB20, CV23, Gongxue, Zhiqiang, Tunyan, Fayin, ST9, LI18, SI17, LI4, ST36, ST40, KI3, LR2, GV15, GV20, CV22, Tounge three needles, Juquan, EX-HN12, EX-HN13	Water-swallowing test, Frenchay dysarthria assessment, ER, symptoms relief time	The total ER (84.00%) of treatment group was significantly higher than that of control group (60.00%) (*P* < 0.05); no adverse events
Xu (2019); RCT	Riluzole 2/d, acupuncture 2/w; 3 m	Riluzole 2/d; 3 m	GV20, GB20, GV14, GB21, BL13, BL15, BL17, BL18, BL20, BL23, GV3, GB30, KI1, GV24, CV22, CV17, LU2, PC3, PC8, CV14, CV12, CV10, CV6, EX-LE2, GB34, LR3, GV16, GV15, EX-B1, GV12, BL42, GV11, BL44, GV9, BL46, GV7, GV4, GV26, ST7, ST6, CV24, CV23, CV22, ST12, CV17, PC1, LI4, SP6	Clinical symptoms, ER	The total ER (95.00%) of treatment group was significantly higher than that of control group (50.00%) (*P* < 0.05)
Li (2019); RCT	Acupoint massage 1/d, Jianpiyifei decoction 2/d, oral Western medicine; 3 m	Oral Western medicine; 3 m	BL43, BL20, BL15, GV12, GV13, EX-B1, BL10, BL23	TCM syndrome score, ALSFRS, Appel scale, EMG	TCM syndrome score ↓, ALSFRS ↑, Apple scale ↓, greater improvement in treatment group (*P* < 0.05); less denervated potentials and simplex patterns of sternocleidomastoid muscle and biceps brachii muscle in treatment group (*p* < 0.05)
Wei (2018); before-and-after study	Electroacupuncture 1/d, moxibustion 1/d; 4 w		EX-B2, CV4, ST36	ALSFRS-R, MMT, ER	ER: 29.40%; ALSFRS-R: 23.70 ± 4.34 → 26.40 ± 4.10 (*P* < 0.05); MMT: 26.47 ± 3.12 → 33.53 ± 3.89 (*P* < 0.01)
Li (2017); RCT	Vitamin, acupuncture 2/d, stellate ganglion block 1/d; 1 m	Vitamin, acupuncture 2/d; 1m	NR	ER, ADL-Barthel index, neurological deficit score	The total ER (80.00%) of treatment group was significantly higher than that of control group (50.00%) (*P* < 0.05); ADL-Barthel: control group: 29.50 ± 3.80 → 39.60 ± 3.10, treatment group: 39.30 ± 3.90 → 51.00 ± 2.60 (*p* < 0.05); neurological deficit score: control group: 21.47 ± 7.14 → 8.60 ± 2.39, treatment group: 21.50 ± 6.83 → 14.00 ± 3.02 (*p* < 0.05); no adverse events
Meng (2017); RCT	Acupuncture 1/d, riluzole 50 mg 2/d; 6 m	Riluzole 50 mg 2/d; 6 m	GV2, GV3, GV4, GV6, GV7, GV8, GV9, GV10, GV11, GV12, GV13, GV14, GV20, GV24	TCM syndrome score, Appel scale, ER	ER: treatment group: 86.70%, control group: 69.20% (*P* < 0.05); TCM syndrome score: treatment group: 5.53 ± 3.14↓, control group: 1.08 ± 3.04↓ (*P* < 0.05); Apple scale: treatment group: 7.08 ± 2.43↓, control group: 5.27 ± 2.09↓ (*P* < 0.05)
Li (2017); RCT	Needle warming through moxibustion 1/d, oral Western medicine; 6 w	Oral Western medicine; 6 w	CV4, CV6, ST36, BL23, BL25	ADL-Barthel index, ER, NDS	ADL-Barthel: treatment group: 30.56 ± 4.32 → 50.36 ± 5.49, control group: 30.53 ± 4.10 → 30.35 ± 6.18 (*P* < 0.05); ER: treatment group: 81.81%, control group: 50.00% (*P* < 0.05); NDS: treatment group: 22.48 ± 7.23 → 8.54 ± 2.38, control group: 22.53 ± 6.73 → 13.80 ± 3.15 (*P* < 0.05)
Sun (2016); RCT	Shenzhejiangqi decoction 3/d, acupuncture 1/d, bloodletting 1/d; 4 w	Shenzhejiangqi decoction 3/d; 4 w	GB20, GV20, CV23, CV12, EX-HN12, EX-HN13	Water-swallowing test, ER	Treatment group: markedly effective: 15, effective: 20, total ER: 87.50%; control group: markedly effective: 7; effective: 14, total ER: 52.50% (*p* < 0.05)
Wu (2016); RCT	Acupuncture, Gegen injection; NR	Swallowing nerve and muscle electrical stimulator 1~2/d; NR	GV20, KI3, GV4, BL20, GB20, LR3, LI10, PC6, CV17, CV4, SP6, CV6, GV14, LI4, LI11, TE5, ST36	Water-swallowing test, ER	Treatment group: cured: 11, effective: 14, total ER: 89.29%; control group: cured: 5, effective: 12, total ER: 60.71% (*p* < 0.05)
Zhang (2014); before-and after-study	Vatimin B1 3/d, Mecobalamin, herbal decoction 2/d, acupuncture 1/d, moxibustion 1/d; 2 m		ST36, LI11, LI4, SP9, GB20, BL20, BL23, GV14, EX-B2, CV6, CV4, GV4, ST40, ST44, SP6	Clinical symptoms, muscle strength, neurological deficit score, sIL-2R, ER	Markedly effective: 18, effective: 12, total ER: 93.75%; neurological deficit score: 13.92 ± 2.10 → 6.33 ± 2.12 (*p* < 0.01); sIL-2R: 289.25 ± 52.18 → 162.16 ± 30.22 (*p* < 0.01)
Zhang (2014); RCT	Acupuncture 1/d, Shenzhejiangqi decoction 2/d, dysphagia therapeutic apparatus 1/d; 4 w	Dysphagia therapeutic apparatus 1/d; 4 w	GV20, CV23, GB20, TE17, GB12, CV12, Juquan, EX-HN12, EX-HN13	Water-swallowing test, ER	ER: 86.67% in treatment group and 43.33% in control group (*P* < 0.05)
Lee (2013); before-and-after study	Sa-am acupuncture 2/d; 5 d		SP3, LU9, HT8, LU10	EtCO_2_, SpO_2_, RR, pulse, K-ALSFRS-R	significant difference in pulse rate and SpO_2_; patients in earlier stages with high K-ALSFRS-R scores responded better to acupuncture treatment
Zhou (2012); RCT	Acupoint massage 1/d, Yifei decoction 2/d, oral Western medicine; 2 w	Oral Western medicine; 2 w	GV12, GV13, BL15, BL20, BL43, EX-B1, BL10, BL23	Body temperature, sputum excretion volume, ventilator parameters, sign, SpO_2_, PaO_2_, PaCO_2_, lung X-ray/CT, WBC, CRP, ER	ER: treatment group: 93.74%; control group: 69.24% (*P* < 0.01); treatment group showed better improvement in sputum excretion volume, ventilator parameters, respiratory function, and blood test (*P* < 0.05); no adverse events
Shen (2012); RCT	Acupoint massage 1/d, Liqitongbian decoction 2/d, oral Western medicine; 2 w	Oral Western medicine; 2 w	CV8, ST25, CV12, CV6, TE6	Stool frequency and character, ventilator parameters, ER	ER: treatment group: 100%; control group: 83.30% (*P* < 0.05); stool character and ventilator parameters improved more in treatment group (*P* < 0.05); 2 cases of diarrhea in treatment group
Chen (2012); RCT	Acupoint injection 1/d, moxibustion 1/d, Shengjizhichan decoction 2/d; 12 w	Riluzole 50mg 2/d; 12 w	PC6, ST36, GV20, GV14, GV6, GV4, GV3, BL-meridian	ER, ALSFRS	ER: treatment group: 66.67%; control group: 36.36% (*P* < 0.05)
Yuan (2005); RCT	Acupuncture 2/d, Jiweiling injection 1/d; 4 w	Qingkailing injection 1/d, oral Western medicine; 4 w	PC6, GV26, GV23, GV20, GV29, GB20, TE17, CV23	Clinical symptoms, ER	ER: treatment group: 88.33%; control group: 5.56% (*P* < 0.01)
Wu (2000); before-and-after study	Acupuncture (shaohuoshan) 1/d in first month, then 1/2d; NR		GV20, BL20, BL23, GV4, KI3, CV17, CV4, CV6, SP6, GV14, LI11, TE5, LI4, ST36, GB20, LI10, PC6, GB34, LR3	Clinical symptoms, ER	Clinically cured: 15
Zhao (1999); before-and-after study	Acupuncture 2/d, scalp acupuncture 2/d; 3 m		PC6, GV26, GV23, GV20, GV29, GB20, GB12, BL10, TE17, CV23, Lower 2/5th motor region	Clinical symptoms, ER	ER: 83.33%

RCT, randomized controlled trial; TCM, traditional Chinese medicine; ER, effective rate; PaO_2_, partial pressure of oxygen; PaCO_2_, partial pressure of carbon dioxide; ALSFRS, Amyotrophic Lateral Sclerosis Functional Rating Scale; EMG, electromyogram; ALSFRS-R, Amyotrophic Lateral Sclerosis Functional Rating Scale-Revised; MMT, Manual Muscle Testing; ADL, Activity of Daily Living; NDS, Neurological Deficiency Score; sIL-2R, soluble interleukin-2 receptor; EtCO_2_, end-tidal carbon dioxide; SpO2, peripheral oxygen saturation; RR, respiratory rate; K-ALSFRS-R, Korean version of the Amyotrophic Lateral Sclerosis Functional Rating Scale-Revised; WBC, white blood cell; CRP, C-reactive protein.

## Discussion

### Summary of findings

Our scoping review examined the breadth and nature of acupuncture use in the treatment of ALS, highlighting gaps in the evidence base. We identified 92 studies that explored the efficacy and safety of acupuncture. We recorded the study design, characteristics of the population, interventions, outcomes, and research findings. Among the included articles, most were conducted in China, and case reports were the most frequently used. Acupuncture interventions included body acupuncture, electroacupuncture, acupoint injection, scalp acupuncture, acupoint massage, Sa-am acupuncture, needle-embedding therapy, auricular acupuncture, pharmacopuncture, bee venom pharmacopuncture therapy, plum blossom needling, acupoint paste, eye acupuncture, fire needle therapy, and needle warming through moxibustion. Acupuncture treatment is often used in combination with herbal medicine or oral Western medicine. The frequency and duration of acupuncture treatment have varied significantly in different studies. Most observational and experimental studies have reported positive results, and adverse events have only been reported in a few studies.

### Gaps in the evidence

#### Too many case study designs

Case reports and case series were the most frequently used study designs included in this scoping review. Only a few RCTs and no systematic reviews have been published to date. Case studies are individualized diagnosis and treatment reports that can provide abundant resources to form various clinical research hypotheses (25). However, there are no control groups in case reports or case series; therefore, we cannot exclude the confounding effect caused by non-research factors when interpreting the research results. As negative results may not be written into a report for submission and publication, there may also be a serious publication bias. Thus, case reports and case series provide relatively limited evidence regarding the application of acupuncture to ALS (26). However, one strength is that the number of RCTs with higher quality evidence has been increasing in recent years.

#### Complex interventions

In the included studies, multiple interventions were often used in combination, such as acupuncture with herbal medicine, acupuncture with oral Western medicine, and acupuncture with other alternative therapies. In addition, the duration and frequency of acupuncture treatments vary significantly. Because ALS is refractory and the efficacy of complementary therapies is uncertain, the combined use of interventions may seem reasonable in clinical practice, but it causes difficulties in the evaluation of efficacy and safety. When used together, it is impossible to evaluate the efficacy of each individual intervention. The diversity of acupuncture interventions also causes confusion among acupuncturists during the treatment process. At the same time, it also brings greater security risks and burdens to patients. Using a single acupuncture method, designing a reasonable acupuncture or control intervention, and selecting the appropriate treatment frequency and duration can more accurately and clearly demonstrate the validity and safety of acupuncture treatment.

#### Limited significance of selected outcomes

Outcomes are closely related to the sample size calculation, description of the results, and interpretation of the research conclusions (27). In the observational studies included, there was an overuse of clinical symptoms as an outcome, whereas in the experimental studies, effective rate was overused. The evaluation of the improvement in clinical symptoms is subjective and vague, and is affected by many factors; therefore, the risk of bias to the research conclusion is large (28). The effective rate is often used to evaluate curative effects in TCM clinical trials. It is a composite index that is not internationally recognized, complicating the interpretation of the results. Excessive use of symptoms and effective rate reduces the reliability of research conclusions (29). At the same time, some earlier articles reported curing ALS. These studies should be treated with caution, as there may be diagnostic errors, data falsification, or other problems. In the study design, internationally recognized outcomes should be selected and standardized according to the purpose of the study to ensure high quality research results and reliable conclusions (30).

Most of the included studies only evaluated short-term and alternative outcomes of acupuncture therapy, such as quality of life or symptom relief, in patients with ALS. Endpoint outcomes, such as survival rate, were not evaluated (31). While the use of alternative or short-term outcomes can greatly reduce the follow-up time and increase the efficiency of the study, focusing on alternative outcomes may lead to failure to observe the true and complete treatment and side effects, resulting in an exaggerated clinical efficacy.

#### Limited study reporting

Although there was no systematic evaluation of study quality in our review, it is easy to find that the reporting of both observational and interventional studies was not exhaustive (32). There were deficiencies in the reporting of demographic information, diagnostic criteria, intervention measures, and adverse events. Moreover, it is important that the reporting of a study remains in accordance with different guidelines, such as the CONSORT or CARE Case Report guidelines to improve the quality of research reports and to enable colleagues to have a deeper understanding of the study design and trust in the results and conclusions of the research (33, 34).

#### Strength and limitations

This review provides a brief summary of the results of the retrieved studies as well as suggestions for future research directions on acupuncture treatment for ALS, which will provide information for health professionals and the research community to adjust and/or develop new research and practices. The intention of a scoping review is simply to summarize the breadth of the available literature, and it refrains from assessing the quality and publication bias of the included studies. Another limitation is that there is obvious heterogeneity in the included articles and this review cannot give answers to more specific questions like a systemic review dose. Due to the large number of studies included, there may be over-simplification of the manipulation of acupuncture methods and findings in the results.

## Conclusion

This scoping review provides a comprehensive overview of the evidence related to acupuncture treatment for ALS. Key evidence gaps include poor study design, complex interventions, limited significance of selected outcomes, and limited study reporting. The promotion of acupuncture treatment for ALS still faces several obstacles. Rigorous study design and conduct, standardized intervention and outcome measurements, and normative reporting are needed to investigate the efficacy and safety of acupuncture treatment for ALS.

## Author contributions

WZ and JN screened the literature. WC and YT extracted data of all the studies. SP wrote the draft of the review. SL and YY revised the manuscript. All authors contributed to the article and approved the submitted version.

## Conflict of interest

The authors declare that the research was conducted in the absence of any commercial or financial relationships that could be construed as a potential conflict of interest.

## Publisher's note

All claims expressed in this article are solely those of the authors and do not necessarily represent those of their affiliated organizations, or those of the publisher, the editors and the reviewers. Any product that may be evaluated in this article, or claim that may be made by its manufacturer, is not guaranteed or endorsed by the publisher.
