# Supplementary material for: Current state of research on acupuncture for the treatment of amyotrophic lateral sclerosis: A scoping review
Source: Front Neurol. 2022 Nov 3;13:1019156. doi: 10.3389/fneur.2022.1019156 (PMC9669253; doi:10.3389/fneur.2022.1019156)
Supplement: Supplementary file 1 [file Table_1.DOCX]

**Search strategy for PubMed**

| No. | Search (untagged = all searchable fields which includes titles, abstracts, and index terms) | Records Retrieved |
| --- | --- | --- |
| 1 | ((((((Acupuncture[MeSH Terms]) OR (Acupuncture Therapy[MeSH Terms])) OR (Electroacupuncture[MeSH Terms])) OR (Acupuncture, Ear[MeSH Terms])) OR (Dry Needling[MeSH Terms])) OR (Acupressure[MeSH Terms])) OR (Acupuncture Points[MeSH Terms]) | 29632 |
| 2 | ((((((((((((Acupuncture) OR (Electroacupuncture)) OR (Pharmacopuncture)) OR (Acupotomy)) OR (Acupotomies)) OR (Acupuncture Point)) OR (Acupoint)) OR (Fire Needle Therapy)) OR (Cutaneous Needle)) OR (Plum Blossom Needle)) OR (Bee Needles)) OR (Catgut Embedment in Acupoint Therapy)) OR (Needle Warming Therapy) | 44297 |
| 3 | 1 OR 2 | 44922 |
| 4 | ((Amyotrophic Lateral Sclerosis[MeSH Terms]) OR (Motor Neuron Disease[MeSH Terms])) OR (Bulbar Palsy, Progressive[MeSH Terms]) | 32576 |
| 5 | (((((((((((((((Amyotrophic Lateral Sclerosis) OR (ALS)) OR (Lou Gehrig's Disease)) OR (Sclerosis, Amyotrophic Lateral)) OR (Guam Disease)) OR (Motor Neuron Disease)) OR (MND)) OR (Neuron Disease, Motor)) OR (Primary Lateral Sclerosis)) OR (PLS)) OR (Anterior Horn Cell Disease)) OR (Progressive Bulbar Palsy)) OR (Progressive Bulbar Paralysis)) OR (PBP)) OR (Progressive Muscular Atrophy)) OR (PMA) | 129050 |
| 6 | 4 OR 5 | 129050 |
| 7 | 3 AND 6 | 147 |

**Search strategy for Cochrane Library**

| No. | (untagged = all searchable fields which includes titles, abstracts, and index terms) | Records Retrieved |
| --- | --- | --- |
| 1# | MeSH descriptor: [Acupuncture] explode all trees | 165 |
| 2# | MeSH descriptor: [Acupuncture Therapy] explode all trees | 5351 |
| 3# | MeSH descriptor: [Electroacupuncture] explode all trees | 896 |
| 4# | MeSH descriptor: [Acupressure] explode all trees | 221 |
| 5# | MeSH descriptor: [Acupressure] explode all trees | 103 |
| 6# | MeSH descriptor: [Acupressure] explode all trees | 429 |
| 7# | MeSH descriptor: [Acupuncture Points] explode all trees | 2276 |
| #8 | (Acupuncture) OR (Electroacupuncture) OR (Pharmacopuncture) OR (Acupotomy) OR (Acupotomies) | 19410 |
| #9 | (Acupuncture Point) OR (Acupoint) OR (Fire Needle Therapy) OR (Cutaneous Needle) OR (Plum Blossom Needle) | 10513 |
| #10 | (Bee Needles) OR (Catgut Embedment in Acupoint Therapy) OR (Needle Warming Therapy) | 373 |
| #11 | #1 OR #2 OR #3 OR #4 OR #5 OR #6 OR #7 OR #8 OR #9 OR #10 | 21734 |
| #12 | MeSH descriptor: [Amyotrophic Lateral Sclerosis] explode all trees | 651 |
| #13 | MeSH descriptor: [Motor Neuron Disease] explode all trees | 815 |
| #14 | MeSH descriptor: [Bulbar Palsy, Progressive] explode all trees | 10 |
| #15 | (Amyotrophic Lateral Sclerosis) OR (ALS) OR (Lou Gehrig's Disease) OR (Sclerosis, Amyotrophic Lateral) OR (Guam Disease) | 100780 |
| #16 | (Motor Neuron Disease) OR (MND) OR (Neuron Disease, Motor) OR (Primary Lateral Sclerosis) OR (PLS) | 5082 |
| #17 | (Anterior Horn Cell Disease) OR (Progressive Bulbar Palsy) OR (Progressive Bulbar Paralysis) OR (PBP) OR (Progressive Muscular Atrophy) | 399 |
| #18 | (PMA) | 888 |
| #19 | #12 OR #13 OR #14 OR #15 OR #16 OR #17 OR #18 | 12431 |
| #20 | #11 AND #19 | 536 |

**Search strategy for WOS**

| No. | TS = title, abstract, author keywords, and Keywords Plus | Records Retrieved |
| --- | --- | --- |
| #1 | (((((((((((((TS=(Electroacupuncture)) OR TS=(Dry Needling)) OR TS=(Acupuncture)) OR TS=(Acupressure)) OR TS=(Pharmacopuncture)) OR TS=(acupotomy)) OR TS=(acupotomies)) OR TS=(Acupoint)) OR TS=(Fire Needle Therapy)) OR TS=(Cutaneous Needle)) OR TS=(Plum Blossom Needle)) OR TS=(Bee Needles)) OR TS=(Catgut Embedment in Acupoint Therapy)) OR TS=(Needle Warming Therapy) | 112411 |
| #2 | (((((((((((TS=(Amyotrophic Lateral Sclerosis)) OR TS=(Motor Neuron Disease)) OR TS=(Bulbar Palsy, Progressive)) OR TS=(ALS)) OR TS=(MND)) OR TS=(PBP)) OR TS=(Primary Lateral Sclerosis)) OR TS=(PLS)) OR TS=(Progressive Bulbar Palsy)) OR TS=(Progressive Muscular Atrophy)) OR TS=(PMA)) OR TS=(Progressive Bulbar Paralysis) | 268260 |
| #3 | #1 AND #2 | 316 |

**Search strategy for CINAHL**

| No. | TX = All text | Records Retrieved |
| --- | --- | --- |
| 1 | TX acupuncture OR TX Electroacupuncture OR TX Dry Needling OR TX Acupressure OR TX Pharmacopuncture OR TX acupotomy OR TX acupotomies OR TX Acupoint OR TX Fire Needle Therapy OR TX Cutaneous Needle OR TX Plum Blossom Needle OR TX Bee Needles OR TX Catgut Embedment in Acupoint Therapy OR TX Needle Warming Therapy | 38595 |
| 2 | TX Amyotrophic Lateral Sclerosis OR TX Motor Neuron Disease OR TX Bulbar Palsy, Progressive OR TX ALS OR TX MND OR TX PBP OR TX Primary Lateral Sclerosis OR TX PLS OR TX Progressive Bulbar Palsy OR TX Progressive Muscular Atrophy OR TX PMA OR TX Progressive Bulbar Paralysis | 45765 |
| 3 | 1 AND 2 | 639 |

**Search strategy for AMED**

| No. | TX = All text | Records Retrieved |
| --- | --- | --- |
| 1 | TX acupuncture OR TX Electroacupuncture OR TX Dry Needling OR TX Acupressure OR TX Pharmacopuncture OR TX acupotomy OR TX acupotomies OR TX Acupoint OR TX Fire Needle Therapy OR TX Cutaneous Needle OR TX Plum Blossom Needle OR TX Bee Needles OR TX Catgut Embedment in Acupoint Therapy OR TX Needle Warming Therapy | 12445 |
| 2 | TX Amyotrophic Lateral Sclerosis OR TX Motor Neuron Disease OR TX Bulbar Palsy, Progressive OR TX ALS OR TX MND OR TX PBP OR TX Primary Lateral Sclerosis OR TX PLS OR TX Progressive Bulbar Palsy OR TX Progressive Muscular Atrophy OR TX PMA OR TX Progressive Bulbar Paralysis | 1095 |
| 3 | 1 AND 2 | 27 |

**Search strategy for CNKI, WanFang, VIP, and CBM**

| Database | Search strategy | Records Retrieved |
| --- | --- | --- |
| CNKI | (SU %= '针刺' OR SU %= '针灸' OR SU %= '针刺治疗' OR SU %= '针刺疗法' OR SU %= '电针' OR SU %= '穴位治疗' OR SU %= '火针' OR SU %= '蜂针' OR SU %= '针刀' OR SU %= '皮内针' OR SU %= '穴位埋线' OR SU %= '温针灸' OR SU %= '梅花针') AND (SU %= '肌萎缩侧索硬化' OR SU %= '运动神经元病' OR SU %= '原发性侧索硬化' OR SU %= '进行性延髓麻痹' OR SU %= '进行性脊肌萎缩') | 125 |
| WanFang | (全部:(肌萎缩侧索硬化) or 全部:(原发性侧索硬化) or 全部:(进行性延髓麻痹) or 全部:(进行性脊肌萎缩) or 全部:(运动神经元病)) and (全部:(针刺) or 全部:(针灸) or 全部:(针刺治疗) or 全部:(针刺疗法) or 全部:(电针) or 全部:(针刀) or 全部:(皮内针) or 全部:(蜂针) or 全部:(穴位埋线) or 全部:(火针) or 全部:(穴位治疗) or 全部:(温针灸) or 全部:(梅花针)) | 375 |
| VIP | U=(肌萎缩侧索硬化 OR 运动神经元病 OR 原发性侧索硬化 OR 进行性延髓麻痹 OR 进行性脊肌萎缩) AND U=(针刺 OR 针灸 OR 针刺疗法 OR 针刺治疗 OR 火针 OR 皮内针 OR 穴位治疗 OR 穴位埋线 OR 温针灸 OR 梅花针 OR 针刀 OR 蜂针) | 160 |
| CBM | (针刺 OR 针灸 OR 针刺疗法 OR 针刺治疗 OR 皮内针 OR 电针 OR 蜂针 OR 穴位治疗 OR 穴位埋线 OR 火针 OR 针刀 OR 梅花针 OR 温针灸) AND (肌萎缩侧索硬化 OR 原发性侧索硬化 OR 进行性延髓麻痹 OR 进行性脊肌萎缩 OR 运动神经元病) | 401 |
