# Supplementary material for: Current state of research on acupuncture for the treatment of amyotrophic lateral sclerosis: A scoping review
Source: Front Neurol. 2022 Nov 3;13:1019156. doi: 10.3389/fneur.2022.1019156 (PMC9669253; doi:10.3389/fneur.2022.1019156)
Supplement: Supplementary file 2 [file Table_2.DOCX]

| Method | Explanation |
| --- | --- |
| Acupuncture | Treatment of disease by inserting needles along specific pathways or meridians. It is sometimes used in conjunction with heat, moxibustion, acupressure, or electric stimulation. |
| Acupoint massage | A type of massage in which finger pressure on specific body sites is used to promote healing and relieve fatigue. The anatomical locations are the same as the acupoints used in acupuncture therapy. |
| Acupoint paste | Acupoint paste is a treatment that works by externally applying herbal paste, which is made from different prescriptions according to treatment purposes, to acupoints. |
| Electroacupuncture | In electroacupuncture therapy, electrical stimulator is connected to the needles after the needling sensation is obtained, and the sensation would be enhanced via the impulsive current to the punctured points. |
| Needle warming through moxibustion | Needle warming through moxibustion is a kind of treatment combining moxibustion and acupuncture, the needle is inserted into the acupoint, and then the moxa stick is burned on the needle handle. The heat is introduced into the acupoint through the needle body, which has both acupuncture effect and warm effect. |
| Sa-am acupuncture | Sa-am acupuncture, an original and traditional Korean acupuncture method, elicits a strong pain response when applied on the upper and lower extremities. Sa-am acupuncture is widely used in Korea. |
| Acupoint injection | Acupoint injection therapy is a method that drug injection is used in acupoints or relevant locations. Exerting a stimulative and pharmaceutical effect on the point via puncture and drug injection. |
| Scalp acupuncture | Scalp acupuncture therapy refers to the therapeutic method of needling the specific areas on the scalp to prevent and treat diseases. The theory of the scalp acupuncture mainly originates from the traditional theories of Zang-fu organs and meridians and collaterals, and the functional areas of the cerebral cortex. |
| Pharmacopuncture | Pharmacopuncture is a new acupuncture treatment in traditional Korean medicine. Pharmacopuncture injects herbal medicine extract at acupoints related to diseases, tender points, or positive reaction points based on meridian theory. |
| Needle-embedding therapy | Needle-embedding therapy implants catgut or other absorbable thread into corresponding acupoints with special needles to produce continuous stimulation. |
| Auricular acupuncture | Auricular acupuncture treats and prevents diseases by stimulating certain points on the auricle with needles or other tools. |
| Eye acupuncture | Eye acupuncture is an understudied intervention and involves fine-needle acupuncture, and may also include embedding catgut at acupoints and acupressure applied around the orbit of the eye. |
| Bee venom pharmacopuncture therapy | Bee venom is a complex mixture of natural products extracted from honey bee which contains various pharmaceutical properties such as peptides, enzymes, and nonpeptide components. The use of bee venom into the specific points is so called bee venom pharmacopuncture therapy. |
| Fire needle therapy | Fire needle therapy is an external treatment method that uses a specific needle that is heated until it burns red and is quickly stabbed into diseased local lesions or acupuncture points. |
| Plum blossom needling | The treatment instrument is made of five or seven stainless-steel needles arranged in a plum-blossom-shaped pattern. Plum-blossom needle therapy treats diseases by tapping specific skin areas or acupoints corresponding to the treatment of different illnesses based on the meridian theory. |
